# Who decides in the household when risk preferences conflict?

**DOI:** 10.1007/s11150-025-09771-8

**Published:** 2025-04-10

**Authors:** Ben D’Exelle, Charlotte Ringdal

**Affiliations:** 1 University of East Anglia, Norwich, UK; 2 Chr. Michelsen Institute and Norwegian School of Economics, Bergen, Norway

**Keywords:** Risk preferences, Household decision-making, Conflict aversion, D1, J12

## Abstract

Women’s low involvement in household decisions is an important cause of the persistence of gender inequality in developing countries, as it constrains women’s access to resources and opportunities. Despite its importance, little is known about the behavioral mechanisms behind women’s low involvement. Guided by a simple theoretical model, we hypothesize that women may refrain from participating in household decisions to avoid spousal conflict, the likelihood of which increases with spousal differences in risk preferences. Using survey data from both spouses of 675 couples in rural Tanzania, we find that spousal differences in risk preferences are associated with a lower likelihood that spouses make decisions jointly and a higher likelihood that decisions are made by the husband alone. These relations are stronger in couples where women are more conflict-averse.

## Introduction

Most women in the world have less favorable access to economic resources and opportunities than men (Klasen, 2020). While some progress has been made in recent decades, large gender inequalities persist. To ensure that gender equality in the world is achieved by 2030, the 5th Sustainable Development Goal of the United Nations (UNDP, 2022), renewed and more effective policy action is needed.

Focusing on household decisions helps us better understand the persistence of gender inequality and potential solutions. Not only are important economic resources allocated within the household; access to household resources is often also essential to access economic opportunities outside the household. This is clearly the case for households in rural Africa, which are the focus of this study.^1^ For example, land is needed to participate in agricultural programs, time and mobility are needed to attend meetings organized by development programs, and access to savings is needed to fund economic investments. It is important to focus on *women’s involvement in household decisions*, as it influences decision outcomes, including women’s access to household resources.^2^

However, little is known about the behavioral mechanisms that influence women’s involvement in household decisions.^3^ Our paper aims to fill this knowledge gap, by testing the hypothesis that spousal preference differences reduce women’s involvement in household decisions. Differences in spousal preferences not only influence the potential impact of women’s involvement on the household decision outcome – if spouses had the same preferences, it would not matter who made the household decision. We will argue that they also directly influence women’s involvement in household decisions. The logic is as follows: We assume that each spouse decides whether to participate in a household decision. If both decide to participate, they need to negotiate on the decision, knowing that this can generate conflict. Spousal preference differences increase the likelihood of spousal conflict, which in turn makes women refrain from participating in household decisions to avoid spousal conflict.

While a range of preferences are relevant for household decisions, we focus on risk preferences for the following reasons: 1) many household decisions involve a degree of risk; 2) men and women often differ in risk preferences (see e.g., Croson and Gneezy (2009)), and assortative matching on risk preferences tends to be low (Di Falco and Vieider, 2018), due to its less observable nature; 3) risk preference heterogeneity can be an important source of conflict (see e.g., Lahno, Serra-Garcia, D’Exelle and Verschoor (2015) and Serra-Garcia (2022)). Using a simple theoretical model, we predict that larger spousal differences in risk preferences decrease the likelihood of joint decision-making and increase the likelihood that decisions are made by the husband alone.

To test this prediction, we use survey data from a sample of 675 couples in rural Tanzania. The survey collected information about risk preferences, conflict aversion, household decision-making and a range of socio-economic characteristics from both spouses of each couple. We find that an increase in risk preference differences is associated with a 21-24 percentage point lower likelihood of household decisions being made jointly, and a 19-21 percentage point higher likelihood that they are made by the husband alone. We also test the prediction that the relation with risk preference differences is stronger among women who face a higher cost of disagreement. Specifically, using women’s conflict aversion as a proxy for the perceived cost of disagreement, we find that the relationship is more pronounced among women who are above the median in conflict aversion. A second proxy for women’s disagreement costs is women’s recent experience of intimate partner violence (IPV). We find that the association between preference differences and decision-making outcomes is not affected by the woman’s experience of IPV.

Our study contributes to the growing economics literature on spousal preferences and intra-household decision-making. It is now commonly accepted that households are characterized by spousal heterogeneity in preferences. We distinguish several strands of literature that investigate how differences in preferences affect household behavior. First, some studies focus on the decision-making outcome. Schaner (2015) shows that couples with different time preferences are more likely to make inefficient savings decisions than couples with similar time preferences. Similarly, Arrondel and Frémeaux (2016) find that risk preference differences have a negative impact on savings and wealth. Serra-Garcia (2022) shows that risk preference differences are associated with a lower production of household public goods and are a predictor of divorce. Second, a few experimental studies analyze the role of spousal preference differences and spousal involvement in decision-making on the outcome of couples’ decisions (Alem, Hassen and Köhlin, 2023; Carlsson, He, Martinsson, Qin and Sutter, 2012, 2013). They find that a couple’s joint decision typically lies between the preferred choice of both spouses, but tends to be closer to the husbands’ preferred choice, confirming husbands’ dominant influence in joint decisions. Third, a few studies document how the disclosure of decisions influences women’s resource allocations within the household (Ashraf, 2009; D’Exelle and Ignowski, 2022). Finally, some studies enrich the literature on spousal preference differences by documenting how spouses often hide resources or income from each other even if this comes at a cost (Almås, Armand, Attanasio and Carneiro, 2018; Anderson and Baland, 2002; Castilla, 2019). Hiding tends to be a common strategy where spousal preferences conflict.

We add to this literature a focus on the mechanisms behind *women’s involvement in household decision-making*, rather than the decision-making outcome. In doing so, we also contribute to the theoretical literature on household decision-making. Cooperative and non-cooperative theoretical models have been developed that model household decision-making.^4^ None of them have looked at the realistic option that where concealing individual actions is not possible, spouses may refrain from being involved in the decision-making process to avoid spousal conflict, which we will do.

The rest of the paper proceeds as follows. Section 2 provides a conceptual framework. Section 3 describes the data. Section 4 presents the results, while Section 5 concludes.

## Conceptual framework

We develop a conceptual framework to formulate testable hypotheses on the effect of preference differences on spousal involvement in household decisions. Consider a household with two spouses *s* ∈ {*w*, *h*}, where *w* represents the wife and *h* represents the husband. Each spouse decides whether to participate in the household decision. They choose the option that maximizes their individual utility *U*_*s*_, which depends on the household decision outcome. This leads to four possible outcomes: both spouses participate (JD), only the wife decides (WD), only the husband decides (HD), or neither decides (ND). The four possibilities with their respective payoff combinations are presented in the game tree in Fig. 1 below.

**Fig. 1:**
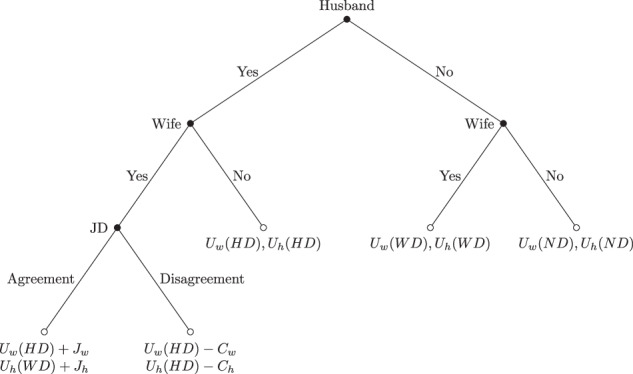
Game tree

We make the following assumptions. First, we assume that preferences about the outcome closely depend on individual risk preferences, and a larger spousal difference in risk preferences leads to a larger spousal difference in preferences about the outcome.^5^ Second, given the patriarchal nature of the society we study (see more on this in our data section), we assume that husbands are first movers. We therefore also assume that if the husband decides not to participate, the wife will make the decision, i.e., *U*_*w*_(*ND*) < *U*_*w*_(*WD*).

If both spouses decide to participate in the decision, an agreement needs to be reached. We assume that *U*_*w*_(*JD*) = *U*_*w*_(*HD*) + *J*_*w*_ with *J*_*w*_ ≥ 0 and *U*_*h*_(*JD*) = *U*_*h*_(*WD*) + *J*_*h*_ with *J*_*h*_ ≥ 0. If they have the same preferences, it would not matter who made the decision, and *J*_*w*_ = 0 and *J*_*h*_ = 0. We have *J*_*w*_ > 0 and *J*_*h*_ > 0, if spouses have different preferences. In other words, an agreement will provide higher utility than if the decision were made by the other spouse.

If they do not reach an agreement, we assume that the husband makes the decision, and there is a cost *C*_*s*_ to both spouses in the form of a deterioration of the spousal relationship. More formally, the utility obtained under joint decision-making has two possible outcomes for each spouse:

1 $${U}_{w}(JD)=\left\{\begin{array}{ll}{U}_{w}(HD)+{J}_{w} \,{\rm{if}}\, {\rm{agreement}}\,\\ {U}_{w}(HD)-{C}_{w}\,{\rm{if}}\, {\rm{disagreement}}\,\end{array}\right.$$

2 $${U}_{h}(JD)=\left\{\begin{array}{ll}{U}_{h}(WD)+{J}_{h}\,{\rm{if}}\, {\rm{agreement}}\,\\ {U}_{h}(HD)-{C}_{h}\,{\rm{if}}\, {\rm{disagreement}}\,\end{array}\right.$$

Before we can find possible equilibria, we need to derive the expected utility obtained under joint decision-making, i.e., *EU*_*w*_(*JD*) and *EU*_*h*_(*JD*). We ignore how a joint decision is made and only assume that there is a probability, *p* ∈ [0, 1], that they will reach an agreement. The expected utility of JD for each spouse would then be:

3 $$E{U}_{w}(JD)=p\left({U}_{w}(HD)+{J}_{w}\right)+(1-p)\left({U}_{w}(HD)-{C}_{w}\right)$$

4 $$E{U}_{h}(JD)=p\left({U}_{h}(WD)+{J}_{h}\right)+(1-p)\left({U}_{h}(HD)-{C}_{h}\right)$$

To determine possible equilibria, we identify the best response of each spouse. First, if the husband participates, the wife’s best response is to participate if *EU*_*w*_(*JD*) > *U*_*w*_(*HD*). Second, if the wife participates, the husband’s best response is to participate if *EU*_*h*_(*JD*) > *U*_*h*_(*WD*). If both conditions hold, JD is an equilibrium.

To examine the effect of spousal preference differences, we assume that *p* decreases with larger preference differences. In other words, the larger the difference in preferences between the husband and the wife, the less likely it is that they will reach an agreement. At the same time, *J*_*w*_ and *J*_*h*_ might also increase with larger preference differences, thereby increasing the first term of equations (3) and (4), i.e., the utility under agreement. We expect the first effect to be stronger than the second, so that the combined effect on *EU*_*w*_ and *EU*_*h*_ will be negative. When the spousal preference difference is sufficiently large, we will have *EU*_*w*_(*JD*) < *U*_*w*_(*HD*). As a result, the wife will prefer not to participate in the decision and let her husband make the decision.

The conceptual framework also shows that *C*_*w*_ contributes to determining when the wife switches from participating in the decision to not participating. It is realistic to assume that *C*_*w*_ is much larger than *C*_*h*_. Husbands tend to have a stronger economic position and often transfer economic resources to their wives. This gives them the option to withhold economic support from their wives. They can also resort to intimate partner violence (IPV). We posit that the wife tends to have little influence on the cost incurred by her husband *C*_*h*_.^6^

With *C*_*w*_ > *C*_*h*_ and the decision being made by the husband if no agreement is reached, disagreement will have a much stronger (negative) effect on *EU*_*w*_(*JD*) than on *EU*_*h*_(*JD*). As a result, an increase in preference differences and the resulting decrease in *p* will induce the wife to step out of the decision before the husband does. This brings us to our first hypothesis.

***Hypothesis 1:*** Larger risk preference differences decrease the likelihood of JD, and increase the likelihood of HD.

The wife’s decision to be involved in the household decision is also influenced by the disagreement cost *C*_*w*_. Additionally, the cost of disagreement may also influence the effect of risk preference differences on the wife’s participation decision. Specifically, if the disagreement cost is very small, then a large risk preference difference is needed to ensure that *EU*_*w*_(*JD*) < *U*_*w*_(*HD*) holds. With a larger disagreement cost, a smaller risk preference difference is needed. This leads to our second hypothesis.

***Hypothesis 2:*** The association between risk preference differences and the likelihood of JD and HD is stronger with larger *C*_*w*_.

## Data

### Setting and sampling

Our study focuses on the Misungwi district, situated 47 kilometers south of Mwanza city in Northern Tanzania, where agriculture serves as the predominant economic activity. This district, characterized by its rural settlements - home to ninety percent of its population (National Bureau of Statistics, 2016) - also engages in livestock keeping, small-scale mining, and the petty trade of agricultural and livestock products. The population is predominantly of the Sukuma tribe, noted for its patrilineal social structure, which typically places decision-making power in the hands of the husbands within households.

For this study, we use survey data collected from a sample of 675 couples across 30 hamlets within the district. Employing a multistage cluster sampling method, eight wards were randomly selected, and from each ward, two villages were chosen at random. Within each village, two hamlets were randomly selected for inclusion in the study. In the selected hamlets, couples were randomly sampled from a complete census of couples where the wife was under 40 years old and had at least one child younger than 12, a criterion reflecting the survey’s broader objective of exploring reproductive health issues.

### Survey data

The survey collected data on, among others, socio-economic characteristics such as age and education, risk preferences, household decision-making, and intimate partner violence. The main variables of interest for our study are the spousal involvement in household decisions, risk preferences, conflict aversion, and intimate partner violence. Below, we describe how each of these variables is measured.

To collect data on women’s involvement in household decision-making, we asked women to what degree they were involved in decisions across six domains: food cooked every day, own health, children’s health, children’s schooling, major household purchases, and visits to friends and family. For each domain, they indicated whether the decisions were normally made by themselves, by the husband, jointly with the husband, or by another person. We exclude the last category, as very few women selected it. In the analysis, we focus on major household purchases, but we also present results for each of the other categories in a separate section. Whether women have a say in major household purchases is commonly used as a measure of empowerment in the literature, as it is thought to be closely related to women’s ability to expand their resource base (Annan, Donald, Goldstein, Martinez and Koolwal, 2021; Donald, Doss, Goldstein and Gupta, 2024; Ting, Ao and Lin, 2014).

The measurement of risk preferences in our study is conducted through a non-incentivized survey question: “Are you generally a person who is fully prepared to take risks or do you avoid taking risks?” Responses are recorded on a scale from 1 to 4, where 1 signifying a strong aversion to risk and 4 indicating a high propensity for risk-taking. Despite the non-incentivized nature of this measure, it has been rigorously validated in the literature as a reliable indicator of risk preferences. Specifically, Dohmen et al. (2011) and Falk et al. (2018) validated it in laboratory settings, and correlations found in other studies with risky behaviors (see e.g., Bonin, Dohmen, Falk, Huffman and Sunde, 2007; Dohmen et al., 2011), underscore its robustness. Dohmen et al. (2011) provide evidence that this survey-based measure is predictive of actual risk-taking behavior. In our analysis, we examine spousal heterogeneity in risk preferences, categorizing the differences into four levels based on the absolute difference in their risk preference scores (0, 1, 2, or 3).

To elicit conflict aversion, we use the questions from the “agreeableness” section of the Big Five questionnaire (Costa and McCrae, 1992): (1) When I have been insulted, I just try to forgive and forget, (2) If someone starts a fight, I am ready to fight back, (3) I hesitate to express my anger even when it is justified, (4) If I do not like people, I let them know it, (5) I sometimes get into arguments, (6) I generally try to be thoughtful and considerate, and (7) I try to be courteous to everyone I meet. For the analysis, we create an index using the first factor of a principal component analysis of the seven questions.

Table 1 provides the descriptive statistics. In terms of demographics, we observe that men in our sample are on average older than women (37 years vs. 31 years). Both men and women have a relatively low level of education with an average of 5 to 5.5 years of schooling. About 38% of women and 30% of men are Catholic, while 30% of women and 44% of men report having no religion. A majority of men (71%) were born in the village in which they currently reside, while only 28% of women reside in the village where they were born. This is in line with the practice of women moving to their husband’s village after marriage. Furthermore, men are more likely to be employed (53% vs. 37%) or own a business (21% vs. 11%).

**Table 1:** Descriptive characteristics

	Wife	Husband	p-value
*Demographics*			
Age	30.8	37.3	0.000
Years of education	5.0	5.4	0.028
Catholic, %	38.1	29.5	0.001
No religion, %	30.4	43.7	0.000
Born in village, %	28.3	71.4	0.000
Employed, %	36.9	53.0	0.000
Own business, %	11.1	20.7	0.000
*Relationship characteristics*			
Relationship length	10.9		
# children	3.1		
Divorced, %	18.2	26.1	0.001
Experienced any IPV, %	55.1		
*Risk preferences and conflict aversion*			
Risk preferences	2.8	3.6	0.000
No difference, %	38.4		
Small difference, %	28.9		
Medium difference, %	11.4		
Large difference, %	21.3		
Conflict aversion	3.9	3.8	0.000
*Who decides on major household purchases*			
Wife decides, %	3.4		
Joint decision, %	60.0		
Husband decides, %	36.6		

The number of observations is 675, with the exception of relationship length (639 observations) and men’s years of education (672 observations). “Employed” takes the value 1 if the respondent reports having worked for someone else in the past 12 months, including on someone else’s farm, business, or for an organization or company (such as school, government and bank). “Own business” takes the value 1 if the respondent reports running a business other than farming (such as a shop, fisher, driver, and builder). “Divorced” takes the value 1 if the respondent reports that they have cohabited or been married to someone else previously. This variable also includes widowers. “Experienced any IPV” takes the value 1 if the wife reports to have experienced economic, psychological, physical, or sexual violence in the past 6 months. Risk preferences are measured on a scale from 1 to 4, where 1 indicates that they avoid risk a lot and 4 indicates that they take risk a lot. Conflict aversion is the average of seven questions ranging from 1 (low degree of conflict aversion) to 5 (high degree of conflict aversion). The answer for each question can be found in Table A2 in Appendix A. The p-value refers to a test of differences of means using a t-test for continuous variables and a proportion test for proportions

Regarding relationship characteristics, we observe that the couples in our sample have, on average, been together for nearly 11 years and have three children. 18% of women and 26% of men have previously cohabited or been married (this includes widowers). 55% of women experienced some kind of intimate partner violence (IPV) in the past six months. See Table A1 in Appendix A for details on the different types of IPV.

In terms of risk preferences, we find, in line with the literature, that women are more risk-averse than men. Still, in 38% of the couples, the spouses have the same risk preferences. In the remaining 62% of the couples, 29%, 11% and 21% display a small, medium, and large difference in preferences, respectively. The distribution of within-couple differences in risk preferences is shown in Fig. A1 in Appendix A. Both women and men display a high degree of conflict aversion with an average score of nearly 4 (out of 5). Answers to the separate questions can be found in Table A2 in Appendix A.

Regarding decisions on major household purchases, as reported by the wife, joint decisions are made in 60% of the couples, while the husband decides in 37% of the couples. The decision-makers in the other domains are shown in Table A3 in Appendix A. In all domains except the food domain, the wife reports joint decision-making in more than 60% of the couples.

## Results

In this section, we investigate the association between spousal risk preference differences and involvement in household decisions. We start by testing Hypothesis 1 with the help of descriptive and regression analysis. Thereafter, we examine the heterogeneity of the association by women’s disagreement costs to test Hypothesis 2. We conclude with some extensions of the analyses and robustness tests.

### Descriptive and regression analysis

**Fig. 2:**
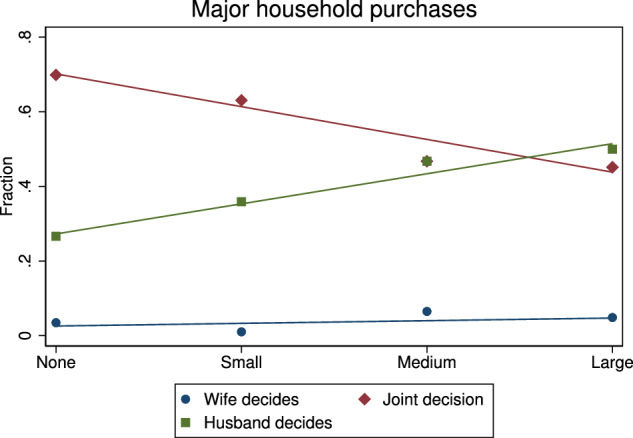
Differences in risk preferences and who decides

We begin with a descriptive analysis of the relationship between spousal differences in risk preferences and decision-making on major household purchases. Figure 2 plots the proportion of couples where the decision is made by the wife only (circles), the husband only (squares), or jointly (rhombuses), for different levels of risk preference differences. We observe that larger differences are associated with a lower likelihood of joint decision-making and a higher likelihood that the decision is made by the husband.^7^ The ANOVA analysis presented in Table A4 in Appendix A shows that these correlations are statistically significant. There is no correlation between risk preference differences and the likelihood that the wife decides alone.

To analyze how spousal risk preference differences relate to the likelihood of a decision is made by the wife, the husband, or jointly, we estimate the following multinomial logit regression where *y*_*i*_ captures the decision-making category as reported by the wife in couple *i*:

$$\begin{array}{rcl}ln\left(\frac{P({y}_{i}=1)}{P({y}_{i}=3)}\right)\,=\,{\beta }_{0}^{1}+{\beta }_{1}^{1}{(\mathrm{Small})}_{i}+{\beta }_{2}^{1}{(\mathrm{Medium})}_{i}+{\beta }_{3}^{1}{(\mathrm{Large})}_{i}+{\beta }_{4}^{1}{X}_{i}+{\varepsilon }_{i}^{1}\\ ln\left(\frac{P({y}_{i}=2)}{P({y}_{i}=3)}\right)\,=\,{\beta }_{0}^{2}+{\beta }_{1}^{2}{(\mathrm{Small})}_{i}+{\beta }_{2}^{2}{(\mathrm{Medium})}_{i}+{\beta }_{3}^{2}{(\mathrm{Large})}_{i}+{\beta }_{4}^{2}{X}_{i}+{\varepsilon }_{i}^{2}\end{array}$$

where (Small)_*i*_, (Medium)_*i*_, and (Large)_*i*_ are indicator variables taking the value 1 if the risk preference difference is small, medium, or large, respectively. The category where the spousal risk preference difference is zero is used as the reference category. *y*_*i*_ is equal to 1 when the wife makes the decision, equal to 2 when the decision is made jointly, and equal to 3 when the decision is made by the husband. *X*_*i*_ is a set of controls, which includes spouses’ religion, education, and employment status, relationship length, whether husband or wife have been divorced before, and whether the wife has experienced any intimate partner violence in the past six months.^8^

Table 2 displays the marginal probabilities for the different risk difference categories relative to no difference in risk preferences. The regression results used to calculate these marginal probabilities are reported in Table B1 in Appendix B. The following insights are worth mentioning. First, in line with the descriptive analysis, the likelihood that the wife decides on major household purchases is not affected by risk preference differences. Second, medium and large differences are associated with a lower likelihood that a decision is made jointly and a higher likelihood that the decision is made by the husband. The effects are substantial. A medium difference in risk preferences, compared to no difference, is associated with a 21.3 - 22.2 percentage point lower probability of a joint decision and an 18.7 - 20.2 percentage point higher probability that the decision is made by the husband. Similarly, a large difference in risk preferences is associated with a 23.5 - 23.9 percentage point lower probability of a joint decision and a 21.2 - 22.8 percentage point higher probability that the decision is made by the husband. Third, a similar pattern is observed for small differences in risk preferences. While the association with a lower probability of the husband deciding is statistically significant, the association with a lower probability of joint decision-making is not (*p* = 0.122 in Column (3) and *p* = 0.153 in Column (4)).

**Table 2:** Risk preference difference and household decision-making on major household purchases

	Wife decides		Joint decision		Husband decides	
	(1)	(2)	(3)	(4)	(5)	(6)
Small difference	−0.042	−0.015	−0.063	−0.063	0.104***	0.078*
	(0.028)	(0.022)	(0.040)	(0.044)	(0.040)	(0.042)
Medium difference	0.021	0.026	−0.222***	−0.213***	0.202***	0.187***
	(0.018)	(0.018)	(0.055)	(0.066)	(0.061)	(0.068)
Large difference	0.011	0.023	−0.239***	−0.235***	0.228***	0.212***
	(0.018)	(0.017)	(0.052)	(0.054)	(0.049)	(0.052)
Observations	675	636	675	636	675	636
Controls	No	Yes	No	Yes	No	Yes

The table displays marginal effects of a multinomial logit model. Outcome variables: Likelihood that the wife makes the decision (Columns (1)-(2)), that the decision is made jointly (Columns (3)-(4)) and that the husband makes the decision (Columns (5)-(6)) regarding major household purchases. Controls: Husband and wife’s age, education, and employment status, whether husband and wife have been divorced previously, relationship length, and whether wife has experienced any IPV. Standard errors clustered at the hamlet level are in parentheses. *p < 0.10, **p < 0.05, ***p < 0.01. See Table B1 in Appendix B for the extended table

The results presented here are in line with Hypothesis 1. We further validate our theoretical assumptions by examining decisions that are relatively trivial for the husband, such as food preparation (see Table A11 in Appendix A). As expected, we find no significant association between spousal risk preference differences and decision-making in this domain, which supports our claim that the observed associations in more consequential decisions reflect the role of risk preferences rather than other factors.

### Heterogeneity: Conflict aversion and intimate partner violence

To test Hypothesis 2, we use two proxies for women’s disagreement costs, *C*_*w*_. A first proxy is women’s conflict aversion. Women who are more conflict-averse face a larger disutility of disagreement. If the influence of risk preference differences on household decision-making is stronger among women who face a higher disagreement cost (as predicted by Hypothesis 2), we should find that the association between risk preference differences and household decision-making is more pronounced among women who are more conflict-averse.

To test this, we interact the risk preference differences with a binary variable equal to one if the wife’s conflict aversion is above the median in the sample, and zero otherwise. Table 3 displays the results. We observe that when the wife is *more* conflict-averse than the median, any difference in risk preferences is associated with a lower likelihood that the decision is made jointly and a higher likelihood that the decision is made by the husband. When the wife is *less* conflict-averse than the median there is no association with who decides. The reported p-values indicate that there are differential associations between couples in which the wife is more conflict-averse than the median and where she is less conflict-averse when the difference is either small or large. While the association goes in the same direction for medium differences, it is not significantly different between the two groups.

**Table 3:** Risk preference difference and household decision-making on major household purchases, by conflict aversion

	Wife decides		Joint decision		Husband decides	
	(1)	(2)	(3)	(4)	(5)	(6)
Small difference, below median	−0.030	−0.004	−0.008	0.006	0.039	−0.002
	(0.021)	(0.020)	(0.063)	(0.073)	(0.063)	(0.070)
Small difference, above median	−0.033***	−0.034***	−0.135***	−0.131**	0.168***	0.165***
	(0.010)	(0.010)	(0.045)	(0.051)	(0.046)	(0.050)
Medium difference, below median	0.016	0.014	−0.150*	−0.141	0.133	0.127
	(0.028)	(0.029)	(0.083)	(0.100)	(0.096)	(0.103)
Medium difference, above median	0.031	0.061	−0.311***	−0.301***	0.280**	0.241**
	(0.046)	(0.052)	(0.089)	(0.093)	(0.110)	(0.114)
Large difference, below median	−0.032	−0.035***	−0.079	−0.067	0.111	0.102
	(0.020)	(0.009)	(0.100)	(0.106)	(0.099)	(0.102)
Large difference, above median	0.045	0.074**	−0.405***	−0.403***	0.360***	0.329***
	(0.032)	(0.037)	(0.069)	(0.069)	(0.078)	(0.080)
Observations	675	636	675	636	675	636
Controls	No	Yes	No	Yes	No	Yes
Below = above, small diff	0.909	0.169	0.104	0.130	0.095	0.054
Below = above, medium diff	0.772	0.414	0.212	0.244	0.340	0.476
Below = above, large diff	0.019	0.003	0.011	0.012	0.080	0.136

The table displays marginal effects of a multinomial logit model. Likelihood that the wife makes the decision (Columns (1)-(2)), that the decision is made jointly (Columns (3)-(4)), and that the husband makes the decision (Columns (5)-(6)) regarding major household purchases. Controls: Husband and wife’s age, education, and employment status, whether husband and wife have been divorced previously, relationship length, and whether wife has experienced any IPV. “Below median” and “Above median” indicate that the conflict aversion of the wife is below or above the median in the sample, respectively. The reported p-values are from a Wald-test, which tests whether the coefficients are the same for women below and above the median. Standard errors clustered at the hamlet level are in parentheses. *p < 0.10, **p < 0.05, ***p < 0.01. See Table A5 for results without margins, and Table B2 for the extended table

A second proxy for women’s disagreement costs is women’s recent experience of intimate partner violence (IPV). While this is an extreme form of conflict that may arise in cases of disagreement, it is very common. As shown in Table 1, 55% of the women report having experienced some form of IPV in the past six months. Assuming that past IPV is a good predictor of future IPV (Oseso et al., 2022), women who recently experienced IPV may have a higher expected cost of disagreement and therefore prefer not to be involved in a household decision, but rather let the husband make the decision.

To test Hypothesis 2, we interact the risk preference differences with a binary variable equal to one if the wife experienced IPV in the last six months, and zero otherwise. Table 4 displays the results. We observe that the recent experience of IPV is not associated with differences in the outcomes when preference differences are large. When preference differences are small or medium, significant associations are found only among women who recently experienced IPV (see Columns (4) and (6)). However, the association is not significantly different between the two groups.^9^ Overall, IPV appears to be an important factor when there are only small differences in risk preferences, but not when the differences are medium or large.

**Table 4:** Risk preference difference and household decision-making on major household purchases, by IPV

	Wife decides		Joint decision		Husband decides	
	(1)	(2)	(3)	(4)	(5)	(6)
Small difference, no IPV	−0.016*	−0.009	−0.004	0.007	0.020	0.002
	(0.010)	(0.007)	(0.063)	(0.069)	(0.062)	(0.069)
Small difference, IPV	−0.036	−0.015	−0.147***	−0.125**	0.183***	0.140**
	(0.023)	(0.029)	(0.049)	(0.053)	(0.059)	(0.055)
Medium difference, no IPV	−0.015*	−0.008	−0.207**	−0.163	0.222**	0.172
	(0.009)	(0.006)	(0.086)	(0.105)	(0.092)	(0.106)
Medium difference, IPV	0.064	0.072	−0.259***	−0.262***	0.195*	0.190*
	(0.047)	(0.058)	(0.078)	(0.088)	(0.105)	(0.113)
Large difference, no IPV	0.021	0.008	−0.280***	−0.253***	0.259***	0.245***
	(0.021)	(0.014)	(0.087)	(0.092)	(0.084)	(0.090)
Large difference, IPV	−0.002	0.036	−0.223***	−0.224***	0.224***	0.188***
	(0.028)	(0.037)	(0.059)	(0.060)	(0.063)	(0.064)
Observations	675	636	675	636	675	636
Controls	No	Yes	No	Yes	No	Yes
No IPV = IPV, small diff	0.426	0.831	0.071	0.115	0.071	0.111
No IPV = IPV, medium diff	0.113	0.182	0.667	0.478	0.854	0.909
No IPV = IPV, large diff	0.527	0.467	0.573	0.774	0.736	0.582

The table displays marginal effects of a multinomial logit model. Likelihood that the wife makes the decision (Columns (1)-(2)), that the decision is made jointly (Columns (3)-(4)), and that the husband makes the decision (Columns (5)-(6)) regarding major household purchases. Controls: Husband and wife’s age, education, and employment status, whether husband and wife have been divorced previously, and relationship length. “No IPV” and “IPV” indicate that the wife has not experienced IPV or has experienced IPV in the past six months. The reported p-values are from a Wald-test, which tests that the coefficients are the same for women who have and who have not experienced IPV. Standard errors clustered at the hamlet level are in parentheses. *p < 0.10, **p < 0.05, ***p < 0.01. See Table A6 for results without margins, and Table B3 for the extended table

### Extensions

In this section, we provide three extensions. First, we investigate whether the relationship between risk preference differences and who is involved in household decision-making extends to other decision-making domains. Table A11 in Appendix A identifies a similar relationship in most other domains, although they are somewhat weaker, as the coefficients are smaller than in Table 2. The only domain where no relation is identified is decisions on the food to prepare everyday. This is not surprising, as these decisions do not involve any risk and are typically made by women following local gender roles.

Second, so far we have used women’s reports about who is involved in household decisions, and it is important to test whether the results hold if we use the husbands’ reports. Previous studies have shown that spouses often disagree on who makes decisions (see e.g., Ambler, Doss, Kieran and Passarelli, 2021, 2022; Anderson, Reynolds and Gugerty, 2017; Annan et al., 2021; Bernard, Doss, Hidrobo, Hoel and Kieran, 2020; Campenhout, Lecoutere and Spielman, 2022). This is also the case in our sample, where spouses agree on who makes the decisions in 48% of the couples. Table A12 in Appendix A displays the association between risk preference differences and household decision-making as reported by the husband. As can be seen, we find no relation between risk preference differences and who decides, when using the husband’s reports. However, the relation is robust when only using the sub-sample of couples where the husband and the wife agree on who makes the decision (see Panel B).

Third, in previous analyses, we used the absolute difference in risk preferences, ignoring which spouse is the more risk-averse. As an extension to our analysis, we investigate whether it matters which spouse is more risk-averse. In 14% of the couples the husband is the more risk-averse spouse, and in 48% the wife is the more risk-averse spouse. Table A13 in Appendix A displays the results. Note that we collapsed large and medium differences for couples where the husband is more risk-averse to ensure that the category is sufficiently large (5.04% of the sample). We find that the associations are primarily observed among couples where the wife is the more risk-averse spouse. Specifically, small, medium, and large differences in risk preferences are associated with a lower likelihood that decisions are made jointly and a higher likelihood that the husband makes the decision. If the husband is the more risk-averse spouse, decisions are more likely to be made jointly and less likely to be made by the wife, but there is no significant association with the likelihood that the husband makes the decision.

### Robustness tests

A potential risk to the identification of the effect of risk preference differences is endogeneity bias, caused by the omission of confounding factors, reverse causality, or survival bias. In this section, we present additional analyses that support the robustness of our results. We start by examining individual risk preferences and other controls, which might be strongly correlated with spousal preference differences and hence confound their estimated effect. Next, we investigate whether time preference differences have a similar effect. Following that, we examine potential reverse causality and survival bias. We conclude this section with regressions that use a control function and a set of instrumental variables to address potential endogeneity bias.

#### Individual risk preferences and other controls

It is plausible that spousal risk preference differences are strongly correlated with individual risk preferences. If individual risk preferences are directly associated with household decisions, the observed association between risk preference differences and household decision-making might be confounded if we do not control for individual risk preferences. To investigate this, we test whether the regression results are robust to the inclusion of individual risk preferences as controls. Table A14 in Appendix A shows that controlling for men’s risk preferences does not change the findings, while controlling for women’s risk preferences does.

This is due to the distribution of risk preferences within our sample. There is limited variation in men’s risk preferences, implying a relatively uniform distribution across the sample. Thus, men’s risk preferences do not significantly contribute to the observed variation in spousal preference differences. In contrast, women’s risk preferences demonstrate a greater degree of variability, thereby becoming a principal driver in the observed differences. In sum, one might argue that our regressions do not fully disentangle the association attributable to spousal preference differences from that linked to women’s individual risk preferences.

To further investigate whether the observed association between spousal risk preference differences and household decision-making might be driven by other observable differences, we re-estimated our models by including an expanded set of control variables. In particular, one might worry that differences in risk preferences are just a proxy for some other spousal difference. To deal with this, we use differences in age, education, religion, conflict aversion, employment status, and business ownership as controls. As shown in Table A15, the inclusion of these additional observable characteristics does not materially affect the magnitude or statistical significance of our key estimates. This robustness check reinforces our conclusion that the association between risk preference differences and household decision-making is not confounded by these observable factors.

#### Time preferences

To provide further evidence supporting the association between spousal preference differences and household decision-making, we analyze a different type of preference, namely time preferences. Our theoretical model is valid for any type of preferences that lead to the same utility differences between the different decision outcomes. Where the time dimension matters for the utility of the decisions – as is the case for decisions on major household purchases – spousal differences in time preferences could have a similar influence on the likelihood of disagreement in JD as risk preference differences.

Time preferences were elicited for a sub-sample (438 out of 675 couples, 65%) during an experiment on family planning decisions (see D’Exelle and Ringdal (2022) for the experiment). Before the main task of the experiment, all participants completed a non-incentivized task to elicit time preferences. We followed the procedure of D’Exelle, van Campenhout and Lecoutere (2012) and asked each participant to make three decisions in private. In each decision, the participants chose between a sooner reward (tomorrow) and a later reward (1 month), where the later reward was always higher than the sooner reward. In the first decision, all participants started by choosing between Tsh 1000 tomorrow (USD 0.45) and Tsh 1500 (USD 0.67) after one month (See Table A16 in Appendix A for details). The participants then received a second decision card where the amount of the later reward was either increased (if they chose the sooner reward in the first decision) or decreased (if they chose the later reward). In the third decision, the participants again received a different later amount, using the same procedure as in the second decision. This elicitation method allows us to categorize the participants into discount factor intervals. We calculate the upper and lower bounds of the intervals as the difference between the sooner and the later options relative to the later option in the third choice, converted into a monthly rate. We then use the midpoint of each interval in the analysis, calculated as the arithmetic mean of the upper and lower bounds. For the last interval, we set the midpoint equal to 23%. Differences in time preferences are measured as the absolute difference between spouses and then standardized. Overall, men and women are equally patient, with a discount factor of about 10%, and an average within-household difference of 8.9 percentage points.

Table A17 in Appendix A displays the results. As can be seen, the results go in the same direction as for risk preferences. When time preference differences increase by one standard deviation, they are associated with a lower likelihood that the decision is made jointly and a higher likelihood that the husband decides. Table A18 in Appendix A includes individual time preferences as controls. We observe that controlling for individual time preferences does not alter the observed association between spousal differences and household decision-making, suggesting that it is indeed the differences in preferences that are most strongly linked to the outcomes.

#### Reverse causality

The wife’s and the husband’s risk preferences may become more similar over time (see, e.g., Di Falco and Vieider, 2018), especially if they make joint decisions. Conversely, they might diverge over time, if there is little interaction between them, i.e., they make decisions individually. If this is the case, part of the estimated effects of risk preference differences in the regressions might be due to reverse causality.

If reverse causality were present, one would expect the observed preference differences to be smaller with increasing duration of the spousal relationship when decisions are made jointly. We examine this relationship for the full sample and for the sub-samples where the decisions are made by the wife, jointly, or by the husband. Table A19 in Appendix A displays the results. If the spouses’ risk preferences become more similar over time, the sign on “Relationship length” should be negative and significant. As we see from the table, the coefficients are positive in the full sample (*p* = 0.020 without controls and *p* = 0.117 with controls), indicating that risk preferences become more dissimilar over time. There is no relationship between the length of the spousal relation and risk preference differences in the sub-samples where the wife or the husband decides. We do find a positive relationship when the decision is made jointly. Specifically, a one-year increase in the duration of the relationship is associated with an increase in the risk preference difference of 0.03 (*p* = 0.009). This result is driven by women becoming more risk-averse the longer they have been in a relationship. Note that this positive correlation goes against the inverse causality mechanism described above, which would require a negative correlation.^10^

#### Survival bias

Over time, some couples divorce, and it is possible that couples currently in a relationship are different from those that dissolved. Importantly, if the correlation between risk preference differences and household decision-making differs between both groups of couples, our results might suffer from ‘survival bias’. It is not immediately clear what the direction of such bias could be. This depends on the correlation between risk preference differences and women’s involvement in household decisions of the dissolved couples. If the correlation between risk preference differences and household decision-making is stronger among couples that dissolved than among those in our sample, our estimates might understate the true association. This could be the case where couples with larger risk preference differences are more likely to experience conflict and get divorced, despite women refraining from being involved in household decisions. Conversely, if women in the couples that divorced refrained less from household decisions than those in our sample, our estimates might overstate the association.

To understand the risk and direction of potential survival bias, we disaggregate our main analysis by the duration of marriage. The regression results with the sub-sample of recently married couples are less vulnerable to survival bias than those for the sample of couples that have been together for a longer time. A comparison between the results across the different sub-samples allows us to get a sense of potential survival bias. Table A20 in Appendix A disaggregates the main regressions. We observe that the results hold for all sub-samples, including the couples that have been together for 13 years or more. This suggests that survival bias is unlikely to be driving the observed associations.

#### Control function

To further assess the robustness of our findings, we estimate new regressions using a control function to account for potential endogeneity bias. Similar to the two-stage least squares approach used in instrumental-variables regression, this approach uses instrumental variables, but instead of replacing the endogenous variable with the prediction from the first stage, it adds the residuals from the first stage. While both approaches lead to the same result with linear models, the consistency of the control function approach is superior with non-linear models (Terza, Basu and Rathouz, 2008). It also has the advantage that the coefficient of the residuals can be used as a heteroskedastic-robust Hausman test of endogeneity (Wooldridge, 2015).

As instruments, we use the weighted average of age, education and risk preferences of the husband and wife’s peers in the village. As peers for the husband, we use all men in the sample who live in the same village, while for women we use all female residents. As weights, we use the pairwise minimum of the number of years of residence in the village of the respondent and the peer, as we assume that the influence of a peer’s characteristics is stronger with more time spent together in the village. We expect that these six instruments are *relevant*, i.e., they influence the risk preferences of husband and wife in a couple, and in this way the risk preference differences in a couple. In addition, regarding the *exclusion restriction*, we do not expect them to have a direct effect on the main dependent variable, i.e., who is involved in the decision, conditional on a set of controls (husband and wife’s age, education, etc.).

In the first stage, we regress risk preference differences on these instruments. The residuals of this control function capture the endogenous part of the risk preference differences, i.e., the variation that is not attributable to the peers’ characteristics. Including these residuals in the second stage regression then addresses potential endogeneity. The estimates of the first-stage control function can be found in Table A21 in Appendix A.

The regression results of the second stage, which includes the residuals of the control function, are presented in Table A22 in Appendix A. The observed association between risk preference differences and household decision-making remains robust when a control function is used, although the estimates are somewhat less efficient. Specifically, a medium difference in risk preferences is associated with a 39.9 percentage point lower likelihood of a joint decision (*p* = 0.069) and a 36.8 percentage point higher likelihood of the husband making the decision (*p* = 0.079). Similarly, a large difference in risk preferences is associated with a 51.9 percentage point lower likelihood of a joint decision (*p* = 0.100) and a 48.7 percentage point higher likelihood of the husband making the decision (*p* = 0.104). The coefficients of the residuals are small and not statistically significant, indicating that endogeneity is unlikely to be driving the observed associations.

## Conclusion

Using survey data from 675 couples in rural Tanzania, we find that spousal differences in risk preferences are associated with who is involved in household decisions. In particular, larger differences are associated with a lower likelihood that the decision is made jointly by both spouses and a higher likelihood that it is made by the husband alone. The association is strongest for major household purchases but is also present in most other domains. These associations are more pronounced in couples where women are more conflict-averse or recently experienced intimate partner violence.

These results align with a theoretical model in which each spouse decides whether to be involved in the household decision. If both decide to be involved in the decision, a joint decision is made, and a negotiation process starts, which might cause disagreement and conflict. Larger risk preference differences increase the likelihood of disagreement, which induces conflict-averse women to refrain from being involved in the household decision.

Two notes are necessary regarding the external validity and policy implications of our findings. First, while our study uses data from only one tribe, we expect our results to extend to other patriarchal societies in Tanzania and East Africa. We believe that it is the patriarchal and inherent gender norms that cause women to face large costs in case of disagreement, the anticipation of which induces women to refrain from being involved in household decisions. This also implies that the results may be very different in matriarchal societies, though such a discussion is outside the scope of our paper. Second, while women’s involvement in household decisions has been shown to be positive for key development outcomes, how policy can influence intra-household decision-making is less clear. Many policy initiatives try to support women’s economic empowerment, assuming it has a positive influence on women’s agency within the household (see e.g., Fiszbein and Schady, 2009). Our results show that less observable and malleable elements, such as spousal preferences, might impose an important limitation on this policy approach. Where women refrain from household decisions due to anticipated conflict, deeper cultural change might be required, such as changes in social norms around the acceptance of intimate partner violence.

## Figures and Tables

**Fig. 1A:**
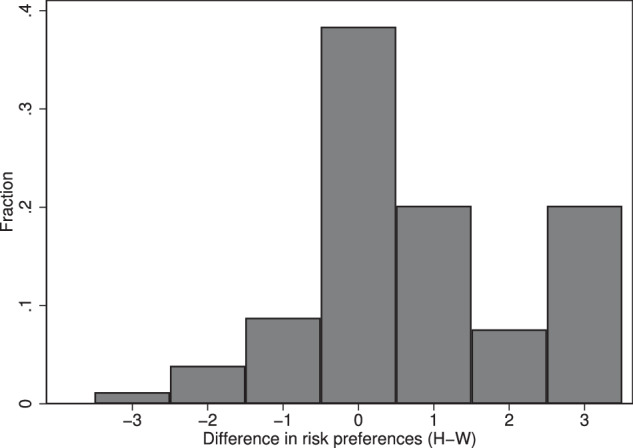
Risk preference differences. Risk preferences are measured on a scale from 1 to 4 where 1 indicates low willingness to take risk and 4 high willingness to take risk. The figure displays the difference between the husband’s and the wife’s risk preferences. Negative values indicate that the wife is more willing to take risk than her husband and positive values that the husband is more willing to take risk than his wife

## Data Availability

Data will be made available upon request.

## Appendix A

Figure 1A, Tables A1–A22.

**Table A1:** Details on intimate partner violence

	Percent
Experienced economic IPV	29.2
Experienced psychological IPV	39.6
Experienced physical IPV	20.0
Experienced sexual IPV	19.0

Economic violence indicates that the husband refused to give the wife enough money for household expenses or if he took away money from her that she had earned. Psychological violence indicates that the husband kept the wife from seeing friends or family, was jealous or angry if she talked to other men, accused her of being unfaithful, humiliated her in front of others or threaten to hurt her or someone close to her. Physical violence is experienced if the husband pushed, shaked, or threw something at the wife, slapped her, punched her, or dragged, kicked or beaten her. Sexual violence indicates that the wife reports that her husband forced her to have sexual intercourse even when she did not want to

**Table A2:** Details on conflict aversion

	Wife	Husband
	Mean	Mean
If someone starts a fight, I’m ready to fight back	4.3	3.8
If I don’t like people, I let them know	4.0	3.5
I sometimes get into arguments	4.0	3.6
When I’ve been insulted I just try to forgive and forget	4.3	4.2
I hesitate to express my anger even when it’s justified	3.3	2.6
I generally try to be thoughtful and considerate	2.0	2.0
I try to be courteous with everyone I meet	1.8	1.9

For all statements on conflict aversion, the scale ranges from 1 to 5 with 1 indicating “strongly agree”, 2 indicating “agree”, 3 indicating “neutral”, 4 indicating “disagree” and 5 indicating “strongly disagree”

**Table A3:** Household decision-making (%)

	Wife	Joint	Husband
Food	78.8	17.0	4.1
Wife’s health	12.2	63.9	23.9
Wife’s mobility	9.6	64.4	26.0
Children’s health	14.3	71.0	14.6
Children’s schooling	5.1	70.7	24.2

**Table A4:** Couple and individual characteristics by risk preference difference

	None	Small	Medium	Large	F-test
*Decision-making*					
Major household purchases	2.23	2.35	2.40	2.45	0.001
Food	1.25	1.23	1.20	1.32	0.307
Wife’s health	2.08	2.11	2.15	2.17	0.468
Children’s health	1.97	2.06	1.95	2.01	0.269
Children’s schooling	2.15	2.20	2.23	2.24	0.280
Wife’s mobility	2.15	2.15	2.23	2.17	0.670
*Wife’s characteristics*					
Age	29.85	31.18	32.55	31.17	0.095
Education	5.16	4.90	5.17	4.79	0.618
Catholic	0.43	0.34	0.31	0.38	0.157
No religion	0.27	0.31	0.34	0.34	0.376
From village	0.25	0.32	0.35	0.26	0.145
Employed	0.38	0.38	0.29	0.38	0.461
Own business	0.14	0.10	0.13	0.06	0.081
Conflict aversion	3.92	3.91	3.98	3.94	0.693
*Husband’s characteristics*					
Age	36.80	37.55	38.83	37.16	0.512
Education	5.71	5.38	4.91	5.01	0.064
Catholic	0.33	0.28	0.23	0.29	0.382
No religion	0.39	0.44	0.52	0.48	0.139
From village	0.72	0.68	0.75	0.73	0.630
Employed	0.50	0.61	0.51	0.50	0.104
Own business	0.20	0.21	0.22	0.20	0.987
Conflict aversion	3.80	3.78	3.80	3.82	0.865
*Couple characteristics*					
Relationship length	9.79	11.26	11.14	12.30	0.029
# children	3.15	3.16	2.95	2.90	0.309
# boys	1.69	1.53	1.62	1.42	0.153
# girls	1.53	1.73	1.43	1.58	0.209
Previously divorced, H	0.27	0.26	0.29	0.23	0.741
Previously divorced, W	0.23	0.12	0.26	0.14	0.004
Wealth index	0.05	−0.11	−0.07	0.19	0.698
Age difference	7.85	7.11	6.83	6.56	0.185
Education difference	2.66	2.60	3.14	2.92	0.443
Different religion	0.51	0.50	0.53	0.53	0.958
Any IPV	0.63	0.49	0.48	0.53	0.017
Economic IPV	0.32	0.29	0.27	0.26	0.627
Psychological IPV	0.44	0.39	0.35	0.34	0.168
Physical IPV	0.24	0.17	0.16	0.19	0.223
Sexual IPV	0.22	0.16	0.26	0.13	0.037

The table displays the mean of each variable for the different levels of risk preference differences together with the p-value from an ANOVA F-test, which tests whether the coefficients are equal across all risk difference levels. The decision-making variables range from 1 to 3 where 1 indicates that the wife makes the decision, 2 that the decision is made jointly and 3 that the husband makes the decision. Conflict aversion ranges from 1 to 5 with higher values indicating higher conflict aversion. ‘Previously divorced’ is an indicator variable equal to 1 if the respondent once divorced from a cohabiting or marriage relation

**Table A5:** Risk preference differences and household decision-making on major household purchases, by conflict aversion

	Wife decides		Joint decision		Husband decides	
	(1)	(2)	(3)	(4)	(5)	(6)
Small difference	−1.135	−0.157	−0.115	0.016	1.135	0.157
	(0.846)	(0.862)	(0.271)	(0.313)	(0.846)	(0.862)
Medium difference	0.069	0.188	−0.600	−0.585	−0.069	−0.188
	(0.761)	(0.956)	(0.383)	(0.440)	(0.761)	(0.956)
Large difference	−1.507	−15.210***	−0.412	−0.370	1.507	15.210***
	(1.139)	(0.599)	(0.418)	(0.460)	(1.139)	(0.592)
Conflict above median	−0.624	−0.407	0.817**	0.864**	0.624	0.407
	(0.994)	(0.879)	(0.389)	(0.406)	(0.994)	(0.879)
Small difference, above median	−13.085***	−14.120***	−0.643*	−0.758*	13.085***	14.120***
	(1.165)	(1.155)	(0.377)	(0.430)	(1.163)	(1.147)
Medium difference, above median	0.219	0.857	−0.817	-0.763	−0.219	−0.857
	(1.364)	(1.396)	(0.657)	(0.689)	(1.364)	(1.396)
Large difference, above median	2.022	16.529***	−1.365**	−1.400**	−2.022	−16.529***
	(1.423)	(1.108)	(0.626)	(0.672)	(1.423)	(1.099)
Constant	−1.861***	−7.357***	0.600***	0.914	1.861***	7.357***
	(0.469)	(1.999)	(0.226)	(0.613)	(0.469)	(1.999)
Observations	675	636	675	636	675	636
Controls	No	Yes	No	Yes	No	Yes

The table displays coefficients from a multinomial logit model. Likelihood that wife makes decision (Columns (1)-(2)), that decision is made jointly (Columns (3)-(4)) and that husband makes decision (Columns (5)-(6)) when it comes to major household purchases. Controls: Husband and wife’s age, education, and employment status, whether husband and wife have been divorced previously, and relationship length. The base category for “wife makes decision” and “joint decision” is “husband makes decision” and the base category for “husband makes decision” is “wife makes decision”. Standard errors clustered at the hamlet level in parenthesis. **p* < 0.10, ***p* < 0.05, ****p* < 0.01

**Table A6:** Risk preference differences and household decision-making on major household purchases, by IPV

	Wife decides		Joint decision		Husband decides	
	(1)	(2)	(3)	(4)	(5)	(6)
Small difference	−13.784***	−12.600***	−0.063	0.007	13.784***	12.600***
	(1.082)	(1.268)	(0.295)	(0.327)	(1.096)	(1.269)
Medium difference	−14.337***	−13.484***	−0.944**	−0.752	14.337***	13.484***
	(1.120)	(1.201)	(0.384)	(0.467)	(1.095)	(1.207)
Large difference	0.791	0.369	−1.169***	−1.099***	−0.791	−0.369
	(1.329)	(1.579)	(0.384)	(0.414)	(1.329)	(1.579)
IPV	1.514	1.672	−0.128	−0.123	−1.514	−1.672
	(1.178)	(1.267)	(0.318)	(0.338)	(1.178)	(1.267)
Small difference, IPV	12.421***	11.908***	−0.683*	−0.618	−12.421***	−11.908***
	(1.394)	(1.348)	(0.396)	(0.402)	(1.401)	(1.349)
Medium difference, IPV	14.817***	14.163***	−0.098	−0.314	−14.817***	−14.163***
	(1.357)	(1.530)	(0.615)	(0.695)	(1.336)	(1.532)
Large difference, IPV	−1.338	−0.130	−0.170	0.149	1.338	0.130
	(1.493)	(1.673)	(0.466)	(0.472)	(1.493)	(1.673)
Constant	−3.219***	−6.911***	1.044***	1.108*	3.219***	6.911***
	(1.058)	(2.060)	(0.242)	(0.638)	(1.058)	(2.060)
Observations	675	636	675	636	675	636
Controls	No	Yes	No	Yes	No	Yes

The table displays coefficients from a multinomial logit model. Likelihood that wife makes decision (Columns (1)-(2)), that decision is made jointly (Columns (3)-(4)) and that husband makes decision (Columns (5)-(6)) when it comes to major household purchases. Controls: Husband and wife’s age, education, and employment status, whether husband and wife have been divorced previously, and relationship length. The base category for “wife makes decision” and “joint decision” is “husband makes decision” and the base category for “husband makes decision” is “wife makes decision”. Standard errors clustered at the hamlet level in parenthesis. **p* < 0.10, ***p* < 0.05, ****p* < 0.01

**Table A7:** Risk preference differences and household decision-making on major household purchases, by economic IPV

	Wife decides		Joint decision		Husband decides	
	(1)	(2)	(3)	(4)	(5)	(6)
Small difference, no IPV	−0.014	−0.006	−0.101**	−0.083	0.115**	0.089
	(0.015)	(0.018)	(0.048)	(0.053)	(0.051)	(0.056)
Small difference, IPV	−0.062**	−0.048	−0.010	0.008	0.072	0.040
	(0.030)	(0.038)	(0.074)	(0.075)	(0.079)	(0.076)
Medium difference, no IPV	−0.022***	−0.019***	−0.221***	−0.191**	0.243***	0.211**
	(0.008)	(0.007)	(0.080)	(0.095)	(0.081)	(0.096)
Medium difference, IPV	0.107*	0.125	−0.264**	−0.275**	0.157	0.150
	(0.064)	(0.085)	(0.109)	(0.111)	(0.123)	(0.133)
Large difference, no IPV	0.027	0.038	−0.285***	−0.279***	0.258***	0.242***
	(0.020)	(0.024)	(0.067)	(0.070)	(0.066)	(0.067)
Large difference, IPV	−0.049	−0.037	−0.165**	−0.136	0.213***	0.174*
	(0.039)	(0.042)	(0.081)	(0.088)	(0.081)	(0.089)
Observations	675	636	675	636	675	636
Controls	No	Yes	No	Yes	No	Yes
No IPV = IPV, small diff	0.166	0.335	0.333	0.321	0.678	0.624
No IPV = IPV, medium diff	0.049	0.093	0.774	0.596	0.576	0.723
No IPV = IPV, large diff	0.098	0.146	0.236	0.183	0.666	0.524

The table displays marginal effects of a multinomial logit model. Likelihood that wife makes decision (Columns (1)-(2)), that decision is made jointly (Columns (3)-(4)) and that husband makes decision (Columns (5)-(6)) when it comes to major household purchases. Controls: Husband and wife’s age, education, and employment status, whether husband and wife have been divorced previously, and relationship length. “No IPV” and “IPV” indicates that the wife has not experienced economic IPV or has experienced economic IPV in the past six months. The reported p-values are from a Wald-test testing that the coefficients are the same for women who have and who have not experienced IPV. Standard errors clustered at the hamlet level in parenthesis. **p* < 0.10, ***p* < 0.05, ****p* < 0.01

**Table A8:** Risk preference differences and household decision-making on major household purchases, by psychological IPV

	Wife decides		Joint decision		Husband decides	
	(1)	(2)	(3)	(4)	(5)	(6)
Small difference, no IPV	−0.025**	−0.021**	−0.007	0.006	0.032	0.014
	(0.010)	(0.009)	(0.045)	(0.052)	(0.044)	(0.051)
Small difference, IPV	−0.034	−0.006	−0.175***	−0.153**	0.209***	0.159**
	(0.028)	(0.033)	(0.062)	(0.064)	(0.067)	(0.067)
Medium difference, no IPV	−0.003	−0.000	−0.235***	−0.195**	0.238***	0.195**
	(0.018)	(0.018)	(0.073)	(0.089)	(0.074)	(0.089)
Medium difference, IPV	0.079	0.065	−0.232***	−0.237**	0.152	0.171
	(0.059)	(0.063)	(0.089)	(0.108)	(0.115)	(0.116)
Large difference, no IPV	0.014	0.009	−0.313***	−0.298***	0.300***	0.289***
	(0.020)	(0.017)	(0.067)	(0.072)	(0.067)	(0.072)
Large difference, IPV	0.006	0.044	−0.141	−0.129	0.135	0.085
	(0.033)	(0.045)	(0.088)	(0.096)	(0.089)	(0.093)
Observations	675	636	675	636	675	636
Controls	No	Yes	No	Yes	No	Yes
No IPV = IPV, small diff	0.767	0.653	0.035	0.059	0.027	0.082
No IPV = IPV, medium diff	0.186	0.301	0.977	0.768	0.513	0.869
No IPV = IPV, large diff	0.832	0.462	0.110	0.150	0.141	0.079

The table displays marginal effects of a multinomial logit model. Likelihood that wife makes decision (Columns (1)-(2)), that decision is made jointly (Columns (3)-(4)) and that husband makes decision (Columns (5)-(6)) when it comes to major household purchases. Controls: Husband and wife’s age, education, and employment status, whether husband and wife have been divorced previously, and relationship length. “No IPV” and “IPV” indicates that the wife has not experienced psychological IPV or has experienced psychological IPV in the past six months. The reported p-values are from a Wald-test testing that the coefficients are the same for women who have and who have not experienced IPV. Standard errors clustered at the hamlet level in parenthesis. **p* < 0.10, ***p* < 0.05, ****p* < 0.01

**Table A9:** Risk preference differences and household decision-making on major household purchases, by physical IPV

	Wife decides		Joint decision		Husband decides	
	(1)	(2)	(3)	(4)	(5)	(6)
Small difference, no IPV	−0.025**	−0.016	−0.065	−0.050	0.091**	0.066
	(0.013)	(0.015)	(0.047)	(0.051)	(0.046)	(0.051)
Small difference, IPV	−0.039	−0.033	−0.129	−0.084	0.168*	0.117
	(0.039)	(0.044)	(0.083)	(0.085)	(0.093)	(0.095)
Medium difference, no IPV	0.002	−0.007	−0.230***	−0.198**	0.228***	0.206***
	(0.024)	(0.018)	(0.070)	(0.081)	(0.073)	(0.079)
Medium difference, IPV	0.116	0.165*	−0.263*	−0.301**	0.146	0.136
	(0.093)	(0.085)	(0.143)	(0.132)	(0.153)	(0.152)
Large difference, no IPV	0.026	0.039	−0.276***	−0.273***	0.250***	0.234***
	(0.024)	(0.026)	(0.058)	(0.061)	(0.053)	(0.056)
Large difference, IPV	−0.076***	−0.083***	−0.146	−0.098	0.222**	0.181
	(0.021)	(0.019)	(0.109)	(0.127)	(0.113)	(0.130)
Observations	675	636	675	636	675	636
Controls	No	Yes	No	Yes	No	Yes
No IPV = IPV, small diff	0.723	0.711	0.517	0.733	0.468	0.651
No IPV = IPV, medium diff	0.251	0.049	0.855	0.530	0.640	0.676
No IPV = IPV, large diff	0.001	0.000	0.245	0.182	0.804	0.688

The table displays marginal effects of a multinomial logit model. Likelihood that wife makes decision (Columns (1)-(2)), that decision is made jointly (Columns (3)-(4)) and that husband makes decision (Columns (5)-(6)) when it comes to major household purchases. Controls: Husband and wife’s age, education, and employment status, whether husband and wife have been divorced previously, and relationship length. “No IPV” and “IPV” indicates that the wife has not experienced physical IPV or has experienced physical IPV in the past six months. The reported p-values are from a Wald-test testing that the coefficients are the same for women who have and who have not experienced IPV. Standard errors clustered at the hamlet level in parenthesis. **p* < 0.10, ***p* < 0.05, ****p* < 0.01

**Table A10:** Risk preference differences and household decision-making on major household purchases, by sexual IPV

	Wife decides		Joint decision		Husband decides	
	(1)	(2)	(3)	(4)	(5)	(6)
Small difference, no IPV	−0.038***	−0.031***	−0.050	−0.031	0.088*	0.062
	(0.010)	(0.008)	(0.044)	(0.054)	(0.047)	(0.055)
Small difference, IPV	0.058	0.067	−0.199**	−0.170**	0.141	0.103
	(0.071)	(0.065)	(0.094)	(0.085)	(0.106)	(0.102)
Medium difference, no IPV	−0.016	−0.011	−0.191***	−0.156*	0.207***	0.167**
	(0.015)	(0.016)	(0.067)	(0.080)	(0.064)	(0.076)
Medium difference, IPV	0.196	0.176	−0.348***	−0.364***	0.151	0.189
	(0.146)	(0.128)	(0.092)	(0.103)	(0.187)	(0.183)
Large difference, no IPV	0.013	0.017	−0.247***	−0.230***	0.234***	0.213***
	(0.017)	(0.017)	(0.066)	(0.074)	(0.063)	(0.072)
Large difference, IPV	−0.052***	−0.057***	−0.229**	−0.210*	0.281**	0.268**
	(0.016)	(0.017)	(0.110)	(0.108)	(0.113)	(0.133)
Observations	670	631	670	631	670	631
Controls	No	Yes	No	Yes	No	Yes
No IPV = IPV, small diff	0.182	0.133	0.148	0.192	0.647	0.743
No IPV = IPV, medium diff	0.155	0.158	0.225	0.144	0.766	0.908
No IPV = IPV, large diff	0.003	0.001	0.894	0.895	0.733	0.738

The table displays marginal effects of a multinomial logit model. Likelihood that wife makes decision (Columns (1)-(2)), that decision is made jointly (Columns (3)-(4)) and that husband makes decision (Columns (5)-(6)) when it comes to major household purchases. Controls: Husband and wife’s age, education, and employment status, whether husband and wife have been divorced previously, and relationship length. “No IPV” and “IPV” indicates that the wife has not experienced sexual IPV or has experienced sexual IPV in the past six months. The reported p-values are from a Wald-test testing that the coefficients are the same for women who have and who have not experienced IPV. Standard errors clustered at the hamlet level in parenthesis. **p* < 0.10, ***p* < 0.05, ****p* < 0.01

**Table A11:** Risk preference differences and household decision-making in other domains

	Panel A: Wife’s health					
	Wife decides		Joint decision		Husband decides	
	(1)	(2)	(3)	(4)	(5)	(6)
Small difference	0.000	−0.010	−0.030	−0.021	0.030	0.031
	(0.029)	(0.031)	(0.034)	(0.036)	(0.029)	(0.032)
Medium difference	0.020	−0.007	−0.106**	−0.077	0.086**	0.084**
	(0.044)	(0.049)	(0.050)	(0.050)	(0.041)	(0.041)
Large difference	0.034	0.027	−0.157***	−0.150**	0.123**	0.122**
	(0.037)	(0.036)	(0.061)	(0.060)	(0.048)	(0.048)
Observations	670	632	670	632	670	632
Controls	No	Yes	No	Yes	No	Yes

	Panel B: Wife’s mobility					
	Wife decides		Joint decision		Husband decides	
	(1)	(2)	(3)	(4)	(5)	(6)
Small difference	−0.011	0.002	0.018	−0.003	−0.007	0.001
	(0.033)	(0.031)	(0.044)	(0.042)	(0.042)	(0.050)
Medium difference	0.024	0.029	−0.127**	− 0.140**	0.103*	0.111*
	(0.037)	(0.037)	(0.064)	(0.067)	(0.054)	(0.057)
Large difference	0.063**	0.062**	−0.163***	− 0.161***	0.100**	0.099**
	(0.026)	(0.027)	(0.047)	(0.049)	(0.042)	(0.048)
Observations	674	635	674	635	674	635
Controls	No	Yes	No	Yes	No	Yes

	Panel C: Children’s health					
	Wife decides		Joint decision		Husband decides	
	(1)	(2)	(3)	(4)	(5)	(6)
Small difference	−0.097**	−0.097*	0.077	0.089	0.020	0.008
	(0.044)	(0.050)	(0.050)	(0.055)	(0.037)	(0.039)
Medium difference	0.049	0.043	−0.086	−0.071	0.038	0.027
	(0.041)	(0.040)	(0.053)	(0.061)	(0.050)	(0.049)
Large difference	0.042	0.059*	−0.121***	−0.127***	0.079*	0.068*
	(0.030)	(0.031)	(0.040)	(0.042)	(0.042)	(0.040)
Observations	669	630	669	630	669	630
Controls	No	Yes	No	Yes	No	Yes

	Panel D: Children’s schooling					
	Wife decides		Joint decision		Husband decides	
	(1)	(2)	(3)	(4)	(5)	(6)
Small difference	−0.021	−0.002	−0.020	−0.029	0.041	0.031
	(0.024)	(0.022)	(0.038)	(0.039)	(0.038)	(0.037)
Medium difference	0.034	0.030	−0.164***	−0.144***	0.130***	0.114**
	(0.023)	(0.023)	(0.048)	(0.051)	(0.049)	(0.051)
Large difference	0.014	0.014	−0.121***	−0.108**	0.106**	0.094*
	(0.020)	(0.021)	(0.041)	(0.045)	(0.043)	(0.048)
Observations	666	628	666	628	666	628
Controls	No	Yes	No	Yes	No	Yes

	Panel E: Which food to prepare every day					
	Wife decides		Joint decision		Husband decides	
	(1)	(2)	(3)	(4)	(5)	(6)
Small difference	0.015	0.018	−0.016	−0.026	0.002	0.008
	(0.040)	(0.041)	(0.038)	(0.039)	(0.017)	(0.020)
Medium difference	0.040	0.041	−0.000	−0.000	−0.040	−0.041
	(0.054)	(0.048)	(0.043)	(0.039)	(0.044)	(0.039)
Large difference	−0.034	−0.053	0.005	0.018	0.029	0.036
	(0.038)	(0.035)	(0.037)	(0.031)	(0.024)	(0.026)
Observations	657	619	657	619	657	619
Controls	No	Yes	No	Yes	No	Yes

*Note:* The table displays marginal effects of a multinominal logit model. Outcome variables: Likelihood that wife makes decision (Columns (1)-(2)), that decision is made jointly (Columns (3)-(4)) and that husband makes decision (Columns (5)-(6)) when it comes to the wife’s health (Panel A), the wife’s mobility (Panel B), Children’s health (Panel C), Children’s schooling (Panel D), and which food to prepare everyday (Panel E). Controls: Husband and wife’s religion, education, and employment status, whether husband and wife have been divorced previously, relationship length, and whether wife has experienced any IPV. Standard errors clustered at the hamlet level in parenthesis. **p* < 0.10, ***p* < 0.05, ****p* < 0.01

**Table A12:** Risk preference differences and household decision-making on major household purchases, husband’s report

	Panel A: Full sample					
	Wife decides		Joint decision		Husband decides	
	(1)	(2)	(3)	(4)	(5)	(6)
Small difference	0.016	0.010	−0.072*	− 0.068	0.056	0.058
	(0.016)	(0.014)	(0.042)	(0.042)	(0.041)	(0.041)
Medium difference	−0.004	−0.007	−0.003	0.017	0.007	−0.010
	(0.027)	(0.023)	(0.079)	(0.077)	(0.073)	(0.073)
Large difference	0.019	0.007	−0.008	0.013	−0.011	−0.020
	(0.018)	(0.023)	(0.053)	(0.052)	(0.049)	(0.057)
Observations	672	633	672	633	672	633
Controls	No	Yes	No	Yes	No	Yes

	Panel B: Sample where husband and wife agree					
	Wife decides		Joint decision		Husband decides	
	(1)	(2)	(3)	(4)	(5)	(6)
Small difference			−0.164***	−0.123*	0.164***	0.123*
			(0.063)	(0.064)	(0.063)	(0.064)
Medium difference			−0.233**	−0.213*	0.233**	0.213*
			(0.113)	(0.114)	(0.113)	(0.114)
Large difference			−0.237***	−0.206**	0.237***	0.206**
			(0.078)	(0.080)	(0.078)	(0.080)
Observations			322	306	322	306
Controls			No	Yes	No	Yes

*Note:* The table displays marginal effects of a multinominal logit model. Outcome variables: Likelihood that wife makes decision (Columns (1)-(2)), that decision is made jointly (Columns (3)-(4)) and that husband makes decision (Columns (5)-(6)). Controls: Husband and wife’s religion, education, and employment status, whether husband and wife have been divorced previously, relationship length, and whether wife has experienced any IPV. Standard errors clustered at the hamlet level in parenthesis. **p* < 0.10, ***p* < 0.05, ****p* < 0.01

**Table A13:** Risk preference differences and household decision-making on major household purchases, by who is more risk averse

	W decides	Joint decision	H decides
*Husband more risk averse*			
Small difference	−0.357***	0.250***	0.107
	(0.058)	(0.075)	(0.077)
Medium - Large difference	0.032	0.063	−0.096
	(0.020)	(0.107)	(0.107)
*Wife more risk averse*			
Small difference	−0.001	−0.118***	0.119***
	(0.021)	(0.044)	(0.042)
Medium difference	0.016	−0.357***	0.341***
	(0.029)	(0.062)	(0.070)
Large difference	0.026	−0.237***	0.212***
	(0.016)	(0.052)	(0.051)
Observations	636	636	636

The table displays marginal effects of a multinominal logit model. Outcome variables: Likelihood that wife makes decision (Column (1)), that decision is made jointly (Column (2)) and that husband makes decision (Column (3)) when it comes to major household purchases. Controls: Husband and wife’s religion, education, and employment status, whether husband and wife have been divorced previously, relationship length, and whether wife has experienced any IPV. Standard errors clustered at the hamlet level in parenthesis. **p* < 0.10, ***p* < 0.05, ****p* < 0.01

**Table A14:** Risk preference differences and household decision-making on major household purchases, with individual risk preferences

	Wife decides		Joint decision		Husband decides	
	(1)	(2)	(3)	(4)	(5)	(6)
Small difference	−0.019	−0.016	0.031	− 0.062	−0.013	0.079*
	(0.024)	(0.024)	(0.053)	(0.046)	(0.050)	(0.044)
Medium difference	0.021	0.022	−0.010	−0.212***	−0.011	0.190***
	(0.027)	(0.024)	(0.071)	(0.066)	(0.075)	(0.073)
Large difference	0.008	0.023	0.137	−0.235***	−0.145	0.212***
	(0.034)	(0.017)	(0.104)	(0.054)	(0.101)	(0.052)
Willingness to take risk, W	−0.006		0.140***		−0.134***	
	(0.012)		(0.029)		(0.029)	
Willingness to take risk, H		−0.003		−0.000		0.003
		(0.010)		(0.031)		(0.031)
Observations	636	636	636	636	636	636

The table displays marginal effects of a multinomial logit model. Likelihood that wife makes decision (Columns (1)-(2)), that decision is made jointly (Columns (3)-(4)) and that husband makes decision (Columns (5)-(6)) about major household purchases. Controls: Husband and wife’s age, education, and employment status, whether husband and wife have been divorced previously, relationship length, and whether wife has experienced any IPV. Standard errors clustered at the hamlet level in parenthesis. **p* < 0.10, ***p* < 0.05, ****p* < 0.01

**Table A15:** Risk preference difference and household decision-making on major household purchases, including different controls

	Wife decides	Joint decision	Husband decides
	(1)	(2)	(3)
Small difference	−0.016	−0.069	0.085**
	(0.022)	(0.042)	(0.040)
Medium difference	0.021	−0.210***	0.189***
	(0.019)	(0.064)	(0.065)
Large difference	0.018	−0.231***	0.213***
	(0.017)	(0.053)	(0.053)
Observations	636	636	636

The table displays marginal effects of a multinomial logit model. Likelihood that wife makes decision (Column (1)), that decision is made jointly (Column (2)) and that husband makes decision (Column (3)) about major household purchases. Controls: Difference in age, education, religion, and conflict aversion, indicator variables taking the value 1 if (i) wife is employed and the husband is not, (ii) husband is employed and the wife is not, (iii) both are employed, (iv) wife owns a business and husband does not, (v) husband owns a business and wife does not, and (vi) both own a business, respectively, whether husband and wife have been divorced previously, duration of relationship, and whether wife has experienced IPV. Standard errors clustered at the hamlet level in parenthesis. **p* < 0.10, ***p* < 0.05, ****p* < 0.01

**Table A16:** Options used in the time preference elicitation task

Choice	Sequence of prior choices	Option A	Option B
1		Tsh 1000	Tsh 1500
2	A	Tsh 1000	Tsh 1750
2	B	Tsh 1000	Tsh 1250
3	AA	Tsh 1000	Tsh 1900
3	AB	Tsh 1000	Tsh 1600
3	BA	Tsh 1000	Tsh 1400
3	BB	Tsh 1000	Tsh 1100

**Table A17:** Time preference differences and household decision-making on major household purchases

	Wife decides		Joint decision		Husband decides	
	(1)	(2)	(3)	(4)	(5)	(6)
Time difference	−0.013	−0.015	−0.033*	−0.031	0.046***	0.046**
	(0.009)	(0.009)	(0.020)	(0.022)	(0.018)	(0.020)
Observations	438	415	438	415	438	415
Controls	No	Yes	No	Yes	No	Yes

The table displays marginal effects of a multinomial logit model. Likelihood that wife makes decision (Columns (1)-(2)), that decision is made jointly (Columns (3)-(4)) and that husband makes decision (Columns (5)-(6)) about major household purchases. The difference in time preferences is standardized. Controls: Husband and wife’s age, education, and employment status, whether husband and wife have been divorced previously, relationship length, and whether wife has experienced any IPV. Standard errors clustered at the hamlet level in parenthesis. **p* < 0.10, ***p* < 0.05, ****p* < 0.01

**Table A18:** Time preference differences and household decision-making on major household purchases with individual time preferences

	Wife decides		Joint decision		Husband decides	
	(1)	(2)	(3)	(4)	(5)	(6)
Difference in time preferences, std	−0.016	−0.019*	−0.025	−0.036	0.041**	0.055**
	(0.010)	(0.010)	(0.023)	(0.024)	(0.021)	(0.022)
Discount rate, W	0.001		−0.005*		0.004*	
	(0.001)		(0.002)		(0.002)	
Discount rate, H		0.001		0.002		−0.004
		(0.001)		(0.003)		(0.003)
Observations	415	415	415	415	415	415

The table displays marginal effects of a multinomial logit model. Likelihood that wife makes decision (Columns (1)-(2)), that decision is made jointly (Columns (3)-(4)) and that husband makes decision (Columns (5)-(6)) about major household purchases. The difference in time preferences is standardized. Controls: Husband and wife’s age, education, and employment status, whether husband and wife have been divorced previously, relationship length, and whether wife has experienced any IPV. Standard errors clustered at the hamlet level in parenthesis. **p* < 0.10, ***p* < 0.05, ****p* < 0.01

**Table A19:** Risk preference differences, by length of relationship

	Full sample		Wife decides		Joint decision			
	(1)	(2)	(3)	(4)	(5)	(6)	(7)	(8)
Relationship length	0.016**	0.017	−0.046	−0.056	0.025***	0.031***	0.006	0.010
	(0.006)	(0.011)	(0.029)	(0.073)	(0.007)	(0.011)	(0.010)	(0.016)
Observations	639	636	20	20	383	383	236	233
Controls	No	Yes	No	Yes	No	Yes		

The table displays OLS regressions. Outcome variables: Absolute difference in risk preferences. Controls: Husband and wife’s age, education, and employment status, whether husband and wife have been divorced previously, relationship length, and whether wife has experienced any IPV. Standard errors clustered at the hamlet level in parenthesis. **p* < 0.10, ***p* < 0.05, ****p* < 0.01

**Table A20:** Risk preference differences and household decision-making on major household purchases, by duration of relationship

	Panel A: Duration of relationship less than 6 years					
	Wife decides		Joint decision		Husband decides	
	(1)	(2)	(3)	(4)	(5)	(6)
Small difference	−0.020	0.005	−0.131**	−0.128**	0.151**	0.123**
	(0.032)	(0.017)	(0.064)	(0.050)	(0.063)	(0.053)
Medium difference	−0.377***	−0.190**	−0.104	−0.213	0.481***	0.402***
	(0.144)	(0.084)	(0.178)	(0.139)	(0.123)	(0.123)
Large difference	0.010	0.046**	−0.264***	−0.275***	0.253***	0.229**
	(0.026)	(0.020)	(0.087)	(0.090)	(0.089)	(0.094)
Observations	203	203	203	203	203	203
Controls	No	Yes	No	Yes	No	Yes

	Panel B: Duration of relationship between 6 and 12 years					
	Wife decides		Joint decision		Husband decides	
	(1)	(2)	(3)	(4)	(5)	(6)
Small difference	−0.568***	−0.447***	0.224*	0.131	0.344***	0.316***
	(0.151)	(0.115)	(0.120)	(0.110)	(0.125)	(0.112)
Medium difference	0.056*	0.065	−0.181*	−0.167	0.125	0.102
	(0.031)	(0.041)	(0.109)	(0.115)	(0.124)	(0.115)
Large difference	0.016	0.045**	−0.252***	−0.295***	0.236**	0.250***
	(0.037)	(0.020)	(0.093)	(0.092)	(0.098)	(0.093)
Observations	209	209	209	209	209	209
Controls	No	Yes	No	Yes	No	Yes

	Panel C: Duration of relationship 13 years or more					
	Wife decides		Joint decision		Husband decides	
	(1)	(2)	(3)	(4)	(5)	(6)
Small difference	−0.023	−0.024	0.021	0.054	0.002	−0.030
	(0.027)	(0.028)	(0.082)	(0.085)	(0.084)	(0.080)
Medium difference	−0.297**	−0.248***	0.085	0.004	0.212**	0.245***
	(0.128)	(0.094)	(0.115)	(0.103)	(0.100)	(0.093)
Large difference	−0.017	−0.020	−0.136**	−0.138*	0.153***	0.158**
	(0.026)	(0.040)	(0.063)	(0.072)	(0.057)	(0.061)
Observations	227	224	227	224	227	224
Controls	No	Yes	No	Yes	No	Yes

The table displays marginal effects of a multinomial logit model. Likelihood that wife makes decision (Columns (1)-(2)), that decision is made jointly (Columns (3)-(4)) and that husband makes decision (Columns (5)-(6)) about major household purchases. Controls: Husband and wife’s age, education, and employment status, whether husband and wife have been divorced previously, and whether wife has experienced any IPV. Standard errors clustered at the hamlet level in parenthesis. **p* < 0.10, ***p* < 0.05, ****p* < 0.01

**Table A21:** Control function - first stage

	Coef.	S.E.	P-value
Mean age of male peers of H	−0.088	(0.027)	0.001
Mean age of female peers of W	0.070	(0.026)	0.008
Proportion of male peers of H completed prim. educ.	−0.603	(0.521)	0.248
Proportion of female peers of W completed prim. educ.	−1.167	(0.559)	0.037
Mean risk preferences of male peers of H	−0.155	(0.266)	0.560
Mean risk preferences of female peers of W	−0.543	(0.214)	0.011
Constant	5.011	(1.351)	0.000

OLS regression. Dependent variable equal to risk preference differences. Averages of peers are weighted by minimum of dyadic number of years of residence in the village. ***, **, * indicate two-sided significance levels at 1, 5, and 10 %, respectively. Standard errors in parentheses

**Table A22:** Risk preference differences and household decision-making on major household purchases, control function

	Wife decides	Joint decision	Husband decides
Small difference	−0.043	−0.152	0.195*
	(0.059)	(0.108)	(0.112)
Medium difference	0.031	−0.399*	0.368*
	(0.091)	(0.219)	(0.210)
Large difference	0.033	−0.519*	0.487
	(0.131)	(0.315)	(0.300)
Residuals	−0.007	0.095	−0.088
	(0.047)	(0.102)	(0.099)
Observations	596	596	596

The table displays marginal effects of a multinominal logit model. Outcome variables: Likelihood that wife makes decision (Column (1)), that decision is made jointly (Column (2)) and that husband makes decision (Column (3)) when it comes to major household purchases. Controls: Control function residual. Standard errors clustered at the hamlet level in parenthesis. **p* < 0.10, ***p* < 0.05, ****p* < 0.01

## Appendix B

Tables B1–B3.

**Table B1:** Risk preference differences and household decision-making on major household purchases, extended

	Wife decides	Joint decision	Husband decides
Small difference	−0.015	−0.063	0.078*
	(0.022)	(0.044)	(0.042)
Medium difference	0.026	−0.213***	0.187***
	(0.018)	(0.066)	(0.068)
Large difference	0.023	−0.235***	0.212***
	(0.017)	(0.054)	(0.052)
Age, wife	0.002	−0.010**	0.008**
	(0.001)	(0.004)	(0.004)
Age, husband	0.001	0.002	−0.003
	(0.001)	(0.004)	(0.004)
Education, wife	−0.003	0.013*	−0.010*
	(0.002)	(0.007)	(0.006)
Education, husband	0.005*	−0.006	0.001
	(0.003)	(0.007)	(0.006)
Is employed, wife	0.000	0.056	−0.057*
	(0.014)	(0.035)	(0.032)
Is employed, husband	−0.023	−0.010	0.033
	(0.016)	(0.039)	(0.038)
Owns business, wife	0.028*	−0.125*	0.096
	(0.016)	(0.066)	(0.066)
Owns business, husband	0.006	−0.037	0.031
	(0.020)	(0.038)	(0.039)
Relationship length	−0.002	0.010**	−0.008*
	(0.001)	(0.005)	(0.005)
Previously divorced, husband	−0.017	0.011	0.007
	(0.015)	(0.056)	(0.057)
Previously divorced, wife	0.028*	0.087	−0.116**
	(0.016)	(0.054)	(0.049)
Experienced any IPV, wife	0.062***	−0.102**	0.040
	(0.021)	(0.041)	(0.034)
Observations	636	636	636

The table displays marginal effects of a multinomial logit model. Outcome variables: Likelihood that wife makes decision (Column (1)), that the decision is made jointly (Column (2)), and that the husband makes the decision (Column (3)) when it comes to major household purchases. Standard errors clustered at the hamlet level in parenthesis. **p* < 0.10, ***p* < 0.05, ****p* < 0.01

**Table B2:** Risk preference difference and household decision-making on major household purchases, by conflict aversion, extended table

	Wife decides	Joint decision	Husband decides
	(1)	(2)	(3)
Small difference, below median	−0.004	0.006	−0.002
	(0.020)	(0.073)	(0.070)
Small difference, above median	−0.034***	−0.131**	0.165***
	(0.010)	(0.051)	(0.050)
Medium difference, below median	0.014	−0.141	0.127
	(0.029)	(0.100)	(0.103)
Medium difference, above median	0.061	−0.301***	0.241**
	(0.052)	(0.093)	(0.114)
Large difference, below median	−0.035***	−0.067	0.102
	(0.009)	(0.106)	(0.106)
Large difference, above median	0.074**	−0.403***	0.329***
	(0.037)	(0.069)	(0.084)
Age wife, below median	0.001	−0.010**	0.009**
	(0.001)	(0.005)	(0.004)
Age wife, above median	0.001	−0.009**	0.008**
	(0.001)	(0.004)	(0.004)
Age husband, below median	0.001	0.002	−0.003
	(0.001)	(0.004)	(0.004)
Age husband, above median	0.001	0.002	−0.003
	(0.001)	(0.003)	(0.003)
Education wife, below median	−0.003	0.013*	−0.010
	(0.002)	(0.007)	(0.006)
Education wife, above median	−0.003	0.012*	−0.009
	(0.002)	(0.006)	(0.005)
Education husband, below median	0.006**	−0.008	0.002
	(0.003)	(0.007)	(0.006)
Education husband, above median	0.006**	−0.007	0.001
	(0.003)	(0.006)	(0.006)
Is employed, wife, below median	−0.025*	−0.013	0.038
	(0.014)	(0.040)	(0.040)
Is employed, wife, above median	−0.025	−0.011	0.036
	(0.019)	(0.039)	(0.035)
Is employed, husband, below median	−0.000	0.071**	−0.071**
	(0.013)	(0.036)	(0.035)
Is employed, husband, above median	0.000	0.062**	−0.062**
	(0.013)	(0.031)	(0.030)
Owns business, wife, below median	0.031**	−0.122*	0.091
	(0.015)	(0.069)	(0.069)
Owns business, wife, above median	0.030*	−0.107*	0.077
	(0.017)	(0.060)	(0.060)
Owns business, husband, below median	0.005	−0.046	0.041
	(0.019)	(0.044)	(0.046)
Owns business, husband, above median	0.005	−0.040	0.035
	(0.018)	(0.040)	(0.042)
Relationship length, below median	−0.001	0.010*	−0.009*
	(0.001)	(0.006)	(0.005)
Relationship length, above median	−0.001	0.009*	−0.008*
	(0.001)	(0.005)	(0.005)
Previously divorced, husband, below median	−0.019	0.009	0.009
	(0.014)	(0.060)	(0.062)
Previously divorced, husband, above median	−0.019	0.008	0.010
	(0.015)	(0.052)	(0.055)
Previously divorced, wife, below median	0.027	0.102*	−0.129**
	(0.017)	(0.060)	(0.055)
Previously divorced, wife, above median	0.028*	0.089*	−0.116**
	(0.016)	(0.052)	(0.048)
Experienced any IPV, below median	0.063**	−0.099**	0.036
	(0.025)	(0.045)	(0.035)
Experienced any IPV, above median	0.062***	−0.088**	0.025
	(0.019)	(0.036)	(0.034)
Observations	636	636	636

The table displays marginal effects of a multinomial logit model. Likelihood that wife makes decision (Column (1)), that decision is made jointly (Column (2)) and that husband makes decision (Column (3)) about major household purchases. “Below median” and “Above median” indicate that the conflict aversion of the wife is below or above the median in the sample, respectively. Standard errors clustered at the hamlet level in parentheses. **p* < 0.10, ***p* < 0.05, ****p* < 0.01

**Table B3:** Risk preference difference and household decision-making on major household purchases, by IPV, extended table

	Wife decides	Joint decision	Husband decides
	(1)	(2)	(3)
Small difference, no IPV	−0.009	0.007	0.002
	(0.007)	(0.069)	(0.069)
Small difference, IPV	−0.015	−0.125**	0.140**
	(0.029)	(0.053)	(0.055)
Medium difference, no IPV	−0.008	−0.163	0.172
	(0.006)	(0.105)	(0.105)
Medium difference, IPV	0.072	−0.262***	0.190*
	(0.058)	(0.088)	(0.114)
Large difference, no IPV	0.008	−0.253***	0.245***
	(0.014)	(0.092)	(0.090)
Large difference, IPV	0.036	−0.224***	0.188***
	(0.037)	(0.060)	(0.064)
Age wife, no IPV	0.000	−0.009**	0.009**
	(0.001)	(0.004)	(0.004)
Age wife, IPV	0.003	−0.011**	0.008**
	(0.002)	(0.005)	(0.004)
Age husband, no IPV	0.000	0.003	−0.003
	(0.000)	(0.004)	(0.003)
Age husband, IPV	0.001	0.002	−0.003
	(0.001)	(0.004)	(0.003)
Education wife, no IPV	−0.001	0.011*	−0.010*
	(0.001)	(0.006)	(0.006)
Education wife, IPV	−0.004	0.013*	−0.009
	(0.004)	(0.007)	(0.006)
Education husband, no IPV	0.001	−0.004	0.002
	(0.001)	(0.006)	(0.006)
Education husband,IPV	0.009*	−0.008	−0.001
	(0.005)	(0.007)	(0.006)
Is employed, wife, no IPV	−0.007	−0.017	0.023
	(0.007)	(0.037)	(0.036)
Is employed, wife, IPV	−0.042	0.002	0.040
	(0.027)	(0.039)	(0.038)
Is employed, husband, no IPV	0.001	0.053	−0.054*
	(0.004)	(0.033)	(0.033)
Is employed, husband, IPV	0.005	0.052	−0.057*
	(0.024)	(0.039)	(0.035)
Owns business, wife, no IPV	0.008	−0.115*	0.107*
	(0.006)	(0.063)	(0.062)
Owns business, wife, IPV	0.050*	−0.142**	0.092
	(0.027)	(0.067)	(0.067)
Owns business, husband, no IPV	0.002	−0.030	0.028
	(0.005)	(0.038)	(0.038)
Owns business, husband, IPV	0.010	−0.035	0.025
	(0.033)	(0.042)	(0.042)
Relationship length, no IPV	−0.000	0.009**	−0.009**
	(0.001)	(0.005)	(0.005)
Relationship length, IPV	−0.003	0.011**	−0.008*
	(0.002)	(0.005)	(0.005)
Previously divorced, husband, no IPV	−0.005	0.006	−0.001
	(0.006)	(0.054)	(0.056)
Previously divorced, husband, IPV	−0.030	0.020	0.010
	(0.025)	(0.057)	(0.060)
Previously divorced, wife, no IPV	0.008	0.097*	−0.105**
	(0.005)	(0.050)	(0.050)
Previously divorced, wife, IPV	0.049*	0.078	−0.127**
	(0.029)	(0.057)	(0.050)
Observations	636	636	636

*Note:* The table displays marginal effects of a multinomial logit model. Likelihood that wife makes decision (Column (1)), that decision is made jointly (Column (2)) and that husband makes decision (Column (3)) about major household purchases. “No IPV” and “IPV” indicate that the wife has not experienced IPV or has experienced IPV in the past six months, respectively. Standard errors clustered at the hamlet level in parenthesis. **p* < 0.10, ***p* < 0.05, ****p* < 0.01

## Footnotes

1 In sub-Saharan Africa, where gender inequality and poverty are particularly severe and intertwined, tackling gender inequality has also an enormous potential to reduce poverty (World Bank, 2012).

2 Recent evidence shows that women’s involvement in household decisions supports key development outcomes such as children’s health and education, and women’s well-being (see e.g., Almås et al., 2022; Duflo, 2012; Ringdal and Sjursen, 2021; Thomas, 1990, 1993), and that the husband has an influence on women’s contraceptive use and fertility (Ashraf, Field and Lee, 2014; D’Exelle and Ringdal, 2022; D’Exelle and Ignowski, 2023), and women’s intra-household allocation (D’Exelle and Ignowski, 2022).

3 For an interesting exception see D’Exelle and Ignowski (2023).

4 Cooperative models assume that the decisions are Pareto efficient, but do not necessarily specify the decision-making process. This category includes the collective model (see e.g., Chiappori, 1988, 1992) and Nash-bargaining models (see e.g., Manser and Brown, 1980; McElroy, 1990; McElroy and Horney, 1981). Non-cooperative models rely on the Cournot-Nash equilibrium where each spouse maximizes their own utility, subject to their own budget constraint, taking the action of the other spouse as given (see e.g., Browning, Chiappori and Lechene, 2010; Ulph, 2006). There are also some models that combine cooperative and non-cooperative bargaining, such as the separate spheres model by Lundberg and Pollak (1993) that start with a cooperative bargaining model and argue that if the bargaining fails a non-cooperative equilibrium emerges.

5 Following this assumption and for ease of explanation, we will use ‘preference differences’ and ‘risk preference differences’ interchangeably in the rest of this section.

6 She can decide to divorce, which might substantially increase the cost for the husband; however, as this would also substantially increase the cost to herself, it would only be used in an extreme case (e.g., after prolonged abuse and when facing risks to her own life). Therefore, this option is not included in the model.

7 Note that the fractions of both categories in Fig. 2 are exactly the same for the ‘medium’ level of risk preference differences.

8 For correlates of risk preference difference categories, see Table A4 in Appendix A.

9 Tables A7–A10 in Appendix A display the results by type of IPV.

10 Note that we do not make any assumptions about the causality between the duration of the marriage and risk preference differences. It could be that couples who have been longer together have had a longer time to influence each other’s risk preferences. It could equally be that risk preference differences influence the likelihood of divorce, which removes these couples from our sample. The key point is to test whether the duration of marriage and risk preference differences are negatively correlated for couples making joint decisions and positively correlated for couples making individual decisions, which they are not.
